# Supplementary material for: The PAS-B Domain of BMAL1 Controls Proliferation, Cellular Energetics, and Inflammatory Response in Human Monocytic Cell Line THP-1
Source: Int J Mol Sci. 2025 Jul 14;26(14):6737. doi: 10.3390/ijms26146737 (PMC12296023; doi:10.3390/ijms26146737)
Supplement: Supplementary file 1 [file ijms-26-06737-s001.zip › IJMS-3736723 Supplemental Fig and Table/Gozu IJMS Figure S1.pptx]

## Slide 1
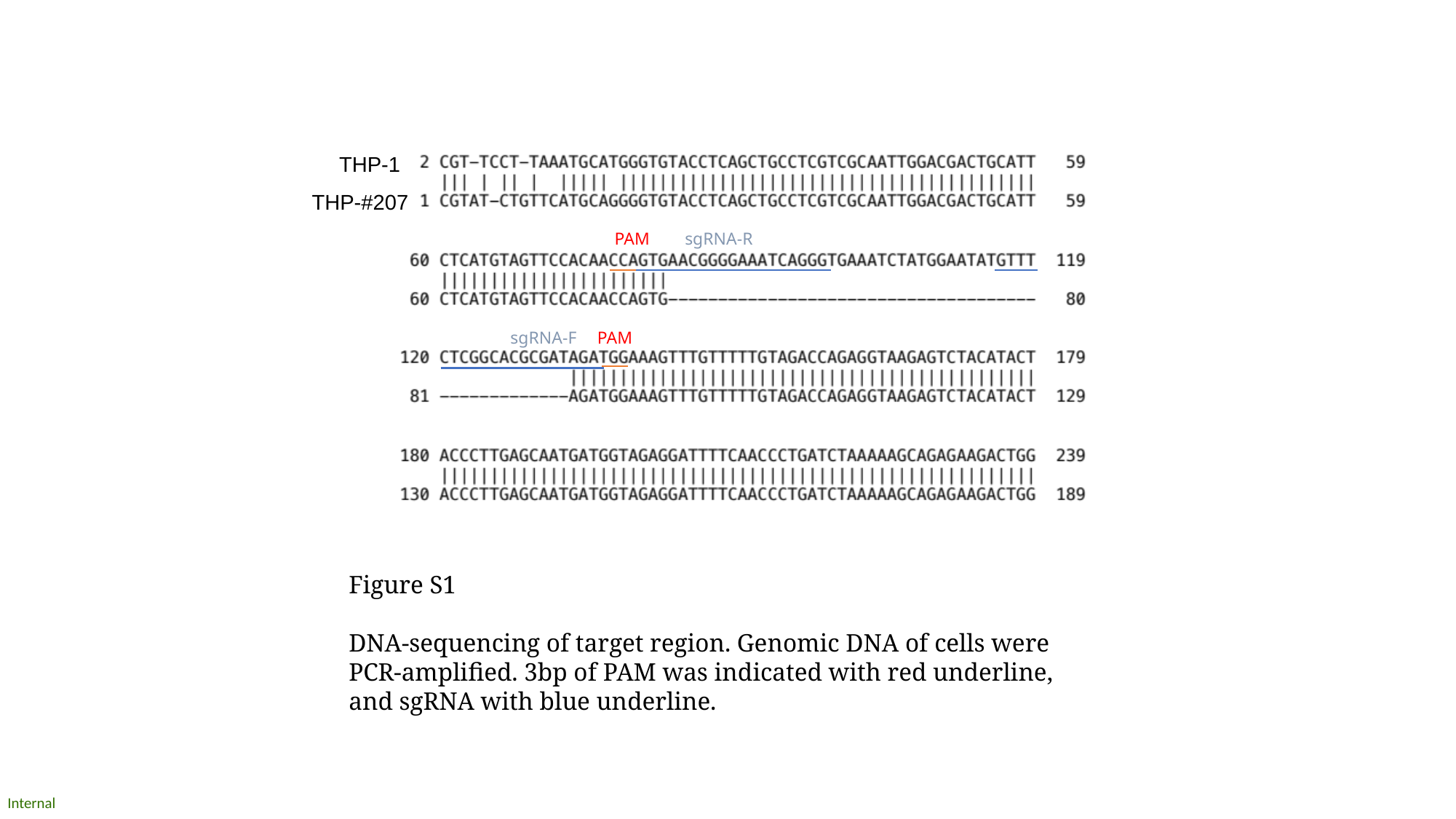

THP-1
THP-#207
PAM
sgRNA-R
PAM
sgRNA-F
Figure S1
DNA-sequencing of target region. Genomic DNA of cells were PCR-amplified. 3bp of PAM was indicated with red underline, and sgRNA with blue underline.
